# Factors associated with the optimal uptake of intermittent preventive treatment in pregnancy with sulfadoxine–pyrimethamine: The experiences of postpartum women attending child welfare clinics in three rural districts in the Western Region of Ghana

**DOI:** 10.1371/journal.pone.0352920

**Published:** 2026-10-27

**Authors:** Gifty Amugi, Daniel Tetteh Agudey, Nana Mireku Gyimah, Pius Mensah, Isaac Kwarkye, Gabriel Yelkoang Gozie Yengliereh, Kofi Jean Claude, Bryony Brookman-Eshun, Sylvester Chinbuah

**Affiliations:** 1 Western Regional Health Directorate, Ghana Health Service, Sekondi-Takoradi, Western Region, Ghana; 2 Mpohor District Health Directorate, Ghana Health Service, Mpohor, Western Region, Ghana; 3 Sekondi-Takoradi Metropolitan Health Directorate, Ghana Health Service, Sekondi-Takoradi, Western Region, Ghana; 4 Jomoro Municipal Health Directorate, Ghana Health Service, Half-Assini, Western Region, Ghana; 5 Sekondi Public Health Laboratory, Sekondi, Western Region, Ghana; Para Federal University, BRAZIL

## Abstract

**Background:**

Intermittent preventive treatment in pregnancy with sulfadoxine–pyrimethamine (IPTp-SP) is a key preventive strategy. However, optimal uptake remains inconsistent despite high antenatal care (ANC) attendance. This study assessed factors associated with optimal IPTp-SP uptake and explored the experiences of postpartum women in rural Ghana.

**Methods:**

A convergent parallel mixed-method study was conducted among 1,155 postpartum women attending child welfare clinics in Jomoro, Prestea-Huni Valley and Ellembelle districts of the Western Region of Ghana. Quantitative data were collected using structured questionnaires and analysed using descriptive statistics and chi-square tests. Qualitative data from in-depth interviews and focus group discussions were analysed thematically to explore women’s experiences and perceptions.

**Results:**

Among the 1,155 women enrolled, 812 (70.3%) took three or more doses of IPTp-SP (IPTp3-SP). Knowledge of malaria in pregnancy was high, with 92.7% correctly identifying its mode of transmission and 91.7% identifying ways of preventing malaria during pregnancy. Factors independently associated with IPTp3-SP included age 10–19 years compared with 20–29 years (aOR 2.17, 95% CI 1.21–3.88), gravidity of three compared with one (aOR 2.67, 95% CI: 1.58–4.50), and gravidity of four compared with one (aOR 1.83, 95% CI: 1.02–3.28). Lower odds were observed among uninsured women (aOR 0.36, 95% CI: 0.20–0.64), single women (aOR 0.64, 95% CI: 0.42–0.97), and cohabiting women (aOR 0.52, 95% CI: 0.34–0.79). ANC attendance showed a strong association with IPTp3-SP: women attending <4 ANC visits had lower odds of receiving IPTp3-SP than those attending 10 or more visits (aOR 0.08, 95% CI: 0.04–0.15). Nausea, dizziness, weakness, abdominal discomfort, vomiting and difficulty chewing the tablets affected their experiences and, for some women, willingness to continue treatment.

**Conclusions:**

IPTp-SP uptake was relatively high. Predictors of optimal uptake include age, education, marital status, gravidity, health insurance status, and number of ANC visits. Women with fewer ANC visits were less likely to receive three or more doses. Reported side effects posed barriers to adherence. Strengthening ANC attendance and counselling on IPTp-SP expected side effects may improve completion of recommended doses.

## Introduction

Malaria remains a major public health problem, particularly in sub–Saharan Africa (SSA). It is caused by protozoan parasites of the genus Plasmodium and transmitted through the bite of an infective female Anopheles mosquito. *Plasmodium falciparum* is responsible for most severe disease and deaths. Globally, an estimated 282 million malaria cases were reported in 2024 across 85 endemic countries, an increase from 227 million cases in 2019, with the majority occurring in the World Health Organization (WHO) African Region [1].

Each year, about 50 million women become pregnant in malaria-endemic countries, and more than half reside in SSA where *P. falciparum* transmission is intense [2,3]. Malaria during pregnancy contributes substantially to maternal and neonatal morbidity and mortality. It is estimated that approximately 10,000 maternal deaths and 200,000 infant deaths occur annually due to malaria infection in pregnancy, largely driven by severe maternal anaemia, low birth weight, and preterm delivery [2,4]. Pregnant women and children under five years remain the most vulnerable groups in endemic settings [1,5].

Ghana is among the countries with stable malaria transmission throughout the year, with seasonal peaks in the northern zones. Malaria accounts for about 38% of outpatient visits, 36% of hospital admissions, and 33% of deaths among children under five years. Among pregnant women, malaria contributes to about 14% of outpatient cases, 11% of hospital admissions, and 9% of maternal deaths [6,7]. *Plasmodium falciparum* accounts for about 90–98% of infections in Ghana, with *P. malariae* and *P. ovale* contributing a smaller proportion [7,8].

Historically, malaria prevention in pregnancy in SSA relied on weekly chloroquine prophylaxis during antenatal care. However, poor adherence and widespread parasite resistance limited its effectiveness. In 2000, the WHO recommended intermittent preventive treatment in pregnancy with sulfadoxine pyrimethamine (IPTp-SP) as a replacement strategy [9]. This strategy, delivered through routine antenatal care services, is one of the three pillars of malaria prevention in pregnancy, alongside insecticide-treated nets and effective case management [1,9].

In 2012, the WHO updated its policy to recommend administration of sulfadoxine pyrimethamine (SP) at each scheduled antenatal care visit starting from the second trimester, with doses given at least one month apart until delivery [10]. Evidence shows that receiving three or more doses is associated with improved birth outcomes and reduced maternal anaemia [11,12].

Despite policy adoption, coverage of IPTp-SP remains suboptimal in many settings. In Ghana, the 2016 Demographic and Health Survey reported that 63% of pregnant women received three or more doses, while 10% received none [13]. In the Western Region, uptake of three or more doses has remained around 50% in recent years, despite high antenatal attendance exceeding 70% for four or more visits. The 2022 Demographic and Health Survey indicated that 49.1% of women in the region received three or more doses during pregnancy [14]. This gap between antenatal attendance and optimal uptake suggests the presence of client level and health system factors that require further examination.

Previous studies in Ghana and other parts of SSA have identified maternal age, education, parity, timing of antenatal initiation, knowledge of malaria in pregnancy, and health system factors as determinants of uptake [7,15–17]. However, limited evidence exists from rural districts in the Western Region that integrates quantitative assessment with lived experiences of postpartum women. Understanding both measurable predictors and experiential factors is essential to guide targeted interventions within routine maternal health services.

This study therefore assessed factors associated with the optimal uptake of IPTp-SP among postpartum women attending child welfare clinics in Jomoro, Prestea-Huni Valley and Ellembelle districts of the Western Region of Ghana. Findings from this study will inform district and regional malaria control efforts aimed at improving protection of pregnant women and newborns in endemic settings.

## Methods

### Study design

The study was conducted using a convergent parallel mixed method approach. Quantitative and qualitative data were collected during the same period and analysed separately, then interpreted together.

### Study setting

The study was conducted in three rural districts in the Western Region of Ghana, namely Ellembelle, Prestea Huni Valley, and Jomoro. These districts are characterized by perennial malaria transmission with seasonal peaks linked to rainfall patterns. The region reports sustained malaria burden among pregnant women despite high antenatal care attendance. District health facilities include hospitals, health centres, and Community based Health Planning and Services compounds that provide routine antenatal and child welfare services.

### Study population

The study population comprised postpartum women who had delivered within three months prior to data collection and were attending child welfare clinics (CWCs) in selected facilities within the three districts.

#### Inclusion criteria.

Women who delivered within three months before the study and attended antenatal care during their most recent pregnancy were eligible.

#### Exclusion criteria.

Women who did not attend antenatal care during pregnancy and those with documented glucose-6-phosphate-dehydrogenase (G6PD) deficiency who were not eligible to receive sulfadoxine pyrimethamine were excluded.

### Sample size determination

For the quantitative component, sample size was estimated using Cochran’s formula for proportions with a 95% confidence level and 5% margin of error [18]. District specific coverage of three [3] or more doses of SP used as the estimated prevalence in Ellembelle, Prestea Huni Valley and Jomoro were 60.6%, 50.8% and 52.9% respectively [14]. The calculated sample sizes were therefore 367 for Ellembelle, 384 for Prestea Huni Valley, and 383 for Jomoro, giving a total sample size of 1134. For the qualitative component, a total of 28 in-depth interviews were conducted with postpartum women. Sample size was guided by the principle of data saturation as described in qualitative research literature [19,20].

### Sampling procedure

A multistage sampling approach was used for the quantitative survey. First, CWCs were selected within each district using simple random sampling. The sampling frame consisted of all functional CWCs in the selected districts, based on updated lists obtained from the respective district health directorates. In the first stage, 15 CWC facilities were selected from 57 functional facilities using simple random sampling in each district. The number of facilities selected from each district was determined using proportional allocation based on the number of functional CWC facilities in each district. Second, systematic sampling was applied on clinic days to recruit eligible women. After selecting the first participant at random, every third eligible woman was invited to participate until the required sample was achieved. A total of 1196 women were approached, of whom 1155 consented and were recruited, giving a response rate of 96.6%. The final quantitative sample comprised 1155 participants, with 374, 391, and 390 recruited from Ellembelle, Prestea Huni Valley, and Jomoro, respectively.

For the qualitative study, purposive sampling was employed to ensure representation of women who completed the at least 3 doses of SP and those who did not. Separate focus group discussions were conducted for women who completed recommended doses and those who did not, to encourage open discussion and minimize social desirability bias. Participants were selected from eligible women who met the study inclusion criteria and were willing to participate in the qualitative component.

The FGDs were conducted by two trained interviewers with experience in qualitative data collection. Before data collection, the interviewers received training on the study objectives, FGD facilitation techniques, use of the discussion guide, informed consent procedures, confidentiality, and management of sensitive or potentially distressing responses. The interviewers were not directly involved in the clinical care of participants, which helped to minimize potential influence on participants’ responses. FGDs were conducted in a private setting at the selected facilities and in a language preferred by the participants.

### Data collection

#### Quantitative data.

Data were collected using a structured interviewer administered questionnaire programmed in Open Data Kit (ODK) in September 2025. The tool captured socio demographic characteristics, antenatal attendance, number of doses received, timing of initiation, knowledge of malaria in pregnancy, and perceived barriers to uptake. The questionnaire was developed in English and administered in English or local languages as appropriate.

The tool was pretested in a district outside the study area using about 10% of the estimated sample size to assess clarity, flow, and internal consistency. Necessary revisions were made before final data collection.

#### Qualitative data.

The qualitative component was designed to provide contextual understanding of the quantitative findings related to IPTp-SP uptake. Data were collected through focus group discussions and in-depth interviews using semi-structured interview guides developed from the study objectives and existing literature on malaria in pregnancy and IPTp-SP uptake. The guides explored women’s experiences with SP administration, perceptions of side effects, understanding of the benefits of IPTp-SP, provider communication, and barriers influencing adherence. Reflexivity was maintained throughout data collection and analysis by considering how professional backgrounds, prior knowledge of the study topic, and how interactions with participants could influence data collection and interpretation. Interviewers were encouraged to use neutral language, avoid leading questions, and allow participants to express their views freely.

Three focus group discussions were conducted among postpartum women, with each group comprising eight participants. Interviews and discussions were conducted in Twi and Nzema. All interviews were audio recorded with participants’ consent. Audio recordings were transcribed verbatim and translated into English. To ensure accuracy and preserve meaning, translated transcripts were independently reviewed and back translated where necessary. Data collection continued until thematic saturation was achieved, defined as the point at which no new themes or insights emerged from subsequent interviews.

### Study variables

The primary outcome variable was uptake of IPTp3-SP, defined as receipt of at least three doses of SP during pregnancy.

Independent variables included age, educational level, occupation, marital status, residence, gravidity, parity, insurance status, gestational age at first dose, number of antenatal visits, knowledge of malaria in pregnancy, availability of SP, and perceived side effects.

### Data analysis

Quantitative data were exported to Statistical Package for Social Sciences version 21 for analysis. Descriptive statistics were computed as frequencies and proportions. Chi-square or Fisher’s exact tests were used to assess associations between categorical independent variables and IPTp3-SP. Multivariable logistic regression was used to identify predictors of IPTp3-SP. Statistical significance was set at p < 0.05.

Qualitative data were analysed using thematic analysis. Transcripts were read repeatedly to achieve familiarization with the data. Thematic analysis was used to identify patterns and themes within the data. An initial coding framework was developed from the study objectives and key areas covered in the FGD guide, while additional codes were generated inductively from participants’ responses. Thematic codes were then applied systematically to the transcripts, and related codes were grouped into broader categories and themes. Codes were independently assigned by two people and differences in coding were discussed until consensus was reached. Coding was conducted manually using Microsoft Excel. Findings were compared across participants who completed the recommended doses and those who did not to identify similarities and differences in their experiences and perceptions.

Credibility of findings was enhanced through investigator review, comparison of responses across participant groups, and triangulation with quantitative findings. Findings were integrated with quantitative results during interpretation.

### Ethical considerations

Ethical approval was obtained from the Ghana Health Service Ethical Review Committee (GHS-ERC:021/03/24). Permission was also obtained from district health directorates and facility managers. Written informed consent was obtained from all participants. For participants who were minors, written informed consent was obtained from their parent or legally authorized guardian, and assent was obtained from the minors before participation. Confidentiality was maintained by using unique identifiers and storing data securely. Participation was voluntary and respondents were free to withdraw at any stage without consequence.

## Results

### Uptake of IPTp-SP by maternal characteristics

Of the 1,155 women analysed, 812 (70.3%) took three or more doses of sulfadoxine-pyrimethamine (SP) during pregnancy (IPTp3-SP). Uptake did not differ significantly by age group (*p* = 0.163). Every other characteristic was associated with uptake in the unadjusted comparison (*p* < 0.001 in each case) (Table 1)

**Table 1:** Uptake of IPTp-SP by maternal characteristics.

Characteristic	Total, n	≥3 doses, n (%)	<3 doses, n (%)	*p* value
**All women**	**1,155**	**812 (70.3)**	**343 (29.7)**	
**Age group (years)**				**0.163**
10–19	91	64 (70.3)	27 (29.7)	
20–29	633	431 (68.1)	202 (31.9)	
30–39	396	294 (74.2)	102 (25.8)	
≥40	33	21 (63.6)	12 (36.4)	
**Educational status**				<0.001*
Primary	420	264 (62.9)	156 (37.1)	
Secondary	431	342 (79.4)	89 (20.6)	
Tertiary	93	84 (90.3)	9 (9.7)	
Not stated	211	122 (57.8)	89 (42.2)	
**Occupation**				<0.001*
Formal employment	112	101 (90.2)	11 (9.8)	
Trading/business	328	259 (79.0)	69 (21.0)	
Farming	306	191 (62.4)	115 (37.6)	
Unemployed/housewife	219	132 (60.3)	87 (39.7)	
Student	99	68 (68.7)	31 (31.3)	
Artisan	91	61 (67.0)	30 (33.0)	
**Marital status**				<0.001*
Married	642	484 (75.4)	158 (24.6)	
Single	305	200 (65.6)	105 (34.4)	
Cohabiting	196	119 (60.7)	77 (39.3)	
Widowed	6	5 (83.3)	1 (16.7)	
Divorced	6	4 (66.7)	2 (33.3)	
**Place of residence**				0.001*
Urban	373	286 (76.7)	87 (23.3)	
Rural	782	526 (67.3)	256 (32.7)	
**Gravidity**				<0.001*
1	339	212 (62.5)	127 (37.5)	
2	327	227 (69.4)	100 (30.6)	
3	215	179 (83.3)	36 (16.7)	
4	137	105 (76.6)	32 (23.4)	
≥5	137	89 (65.0)	48 (35.0)	
**Health insurance**				<0.001*
Insured	1,083	783 (72.3)	300 (27.7)	
Not insured	72	29 (40.3)	43 (59.7)	
**Gestational age at booking (weeks)**				<0.001*
<12	405	304 (75.1)	101 (24.9)	
12–16	454	335 (73.8)	119 (26.2)	
17–21	112	67 (59.8)	45 (40.2)	
21–24	29	15 (51.7)	14 (48.3)	
>24	155	91 (58.7)	64 (41.3)	
**Number of ANC visits**				<0.001*
<4	181	66 (36.5)	115 (63.5)	
4–6	497	330 (66.4)	167 (33.6)	
7–9	328	282 (86.0)	46 (14.0)	
≥10	149	134 (89.9)	15 (10.1)	

*p < 0.05. ANC, antenatal care.

### The proportion of postpartum women who took IPTp during pregnancy

About 95.7% (1105/1155) of respondents reported taking at least one dose of IPTp-SP during pregnancy (Fig 1).

**Fig 1:**
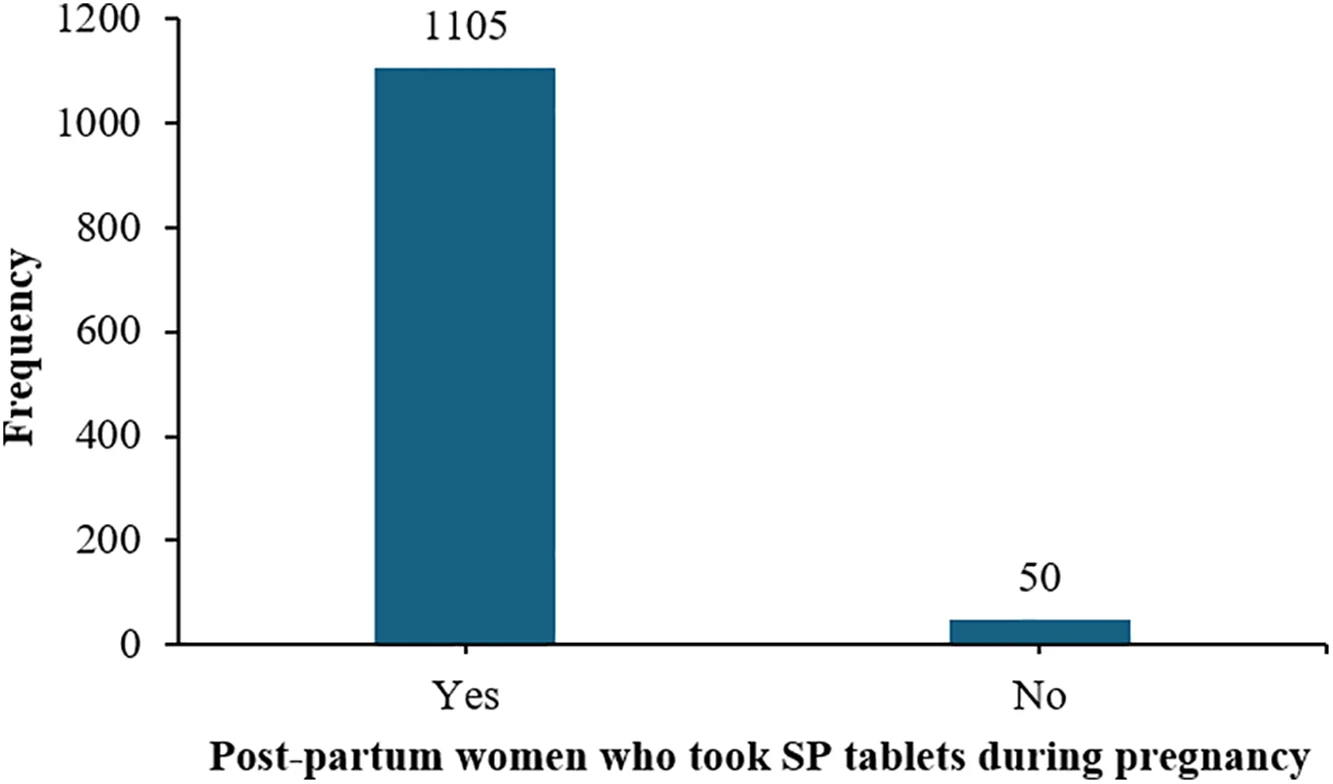
Postpartum women who took IPTp during pregnancy.

### The level of knowledge of Postpartum Women on Malaria in Pregnancy (MIP) And IPTP-SP

Table 2 shows the knowledge of respondents on malaria in pregnancy and IPTp-SP. It was found that 92.7% (1027/1155) of respondents have knowledge on how malaria is transmitted in pregnancy, 75.1% (830/1155) had knowledge of the effect of malaria on pregnancy, 83.5% (923/1155) had knowledge on the effect of malaria infection on the foetus and 91.7% (1013/1155) had knowledge of prevention of malaria infection in pregnancy.

**Table 2:** Knowledge Of Postpartum Women on MIP And IPTP-SP.

Knowledge of MIP	Yes (n)	%	No (n)	%
Knowledge of how malaria is transmitted in pregnancy.	1027	92.9%	78	7.1%
Knowledge of the effect of malaria on pregnancy.	830	75.1%	275	24.9%
Knowledge of the effect of malaria infection on the fetus.	923	83.5%	182	16.5%
Knowledge of prevention of malaria infection in pregnancy.	1013	91.7%	92	8.3%

### Causes of malaria

About 85.4% (986/1155) of participants identified mosquito bites as the main cause, while only a small number attributed malaria to incorrect causes such as exposure to heat or drinking dirty water, and some reported having no idea (Fig 2).

**Fig 2:**
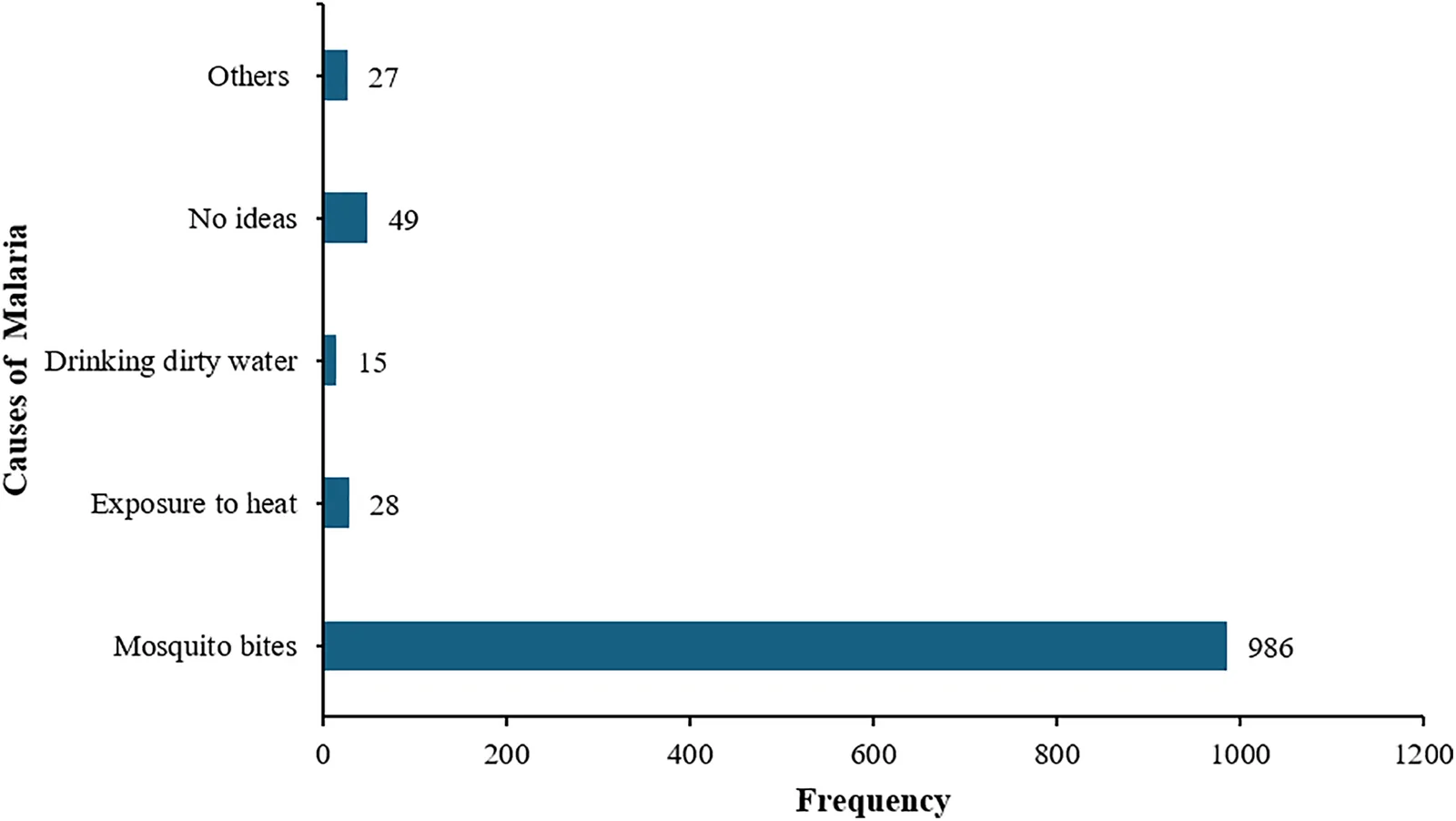
Level of knowledge on the Causes of Malaria among Postpartum Women.

### Factors affecting the uptake of SP

Side effects associated with SP were the most frequently reported factor influencing the uptake of IPTp SP among respondents (64%, 32/50) (Fig 3).

**Fig 3:**
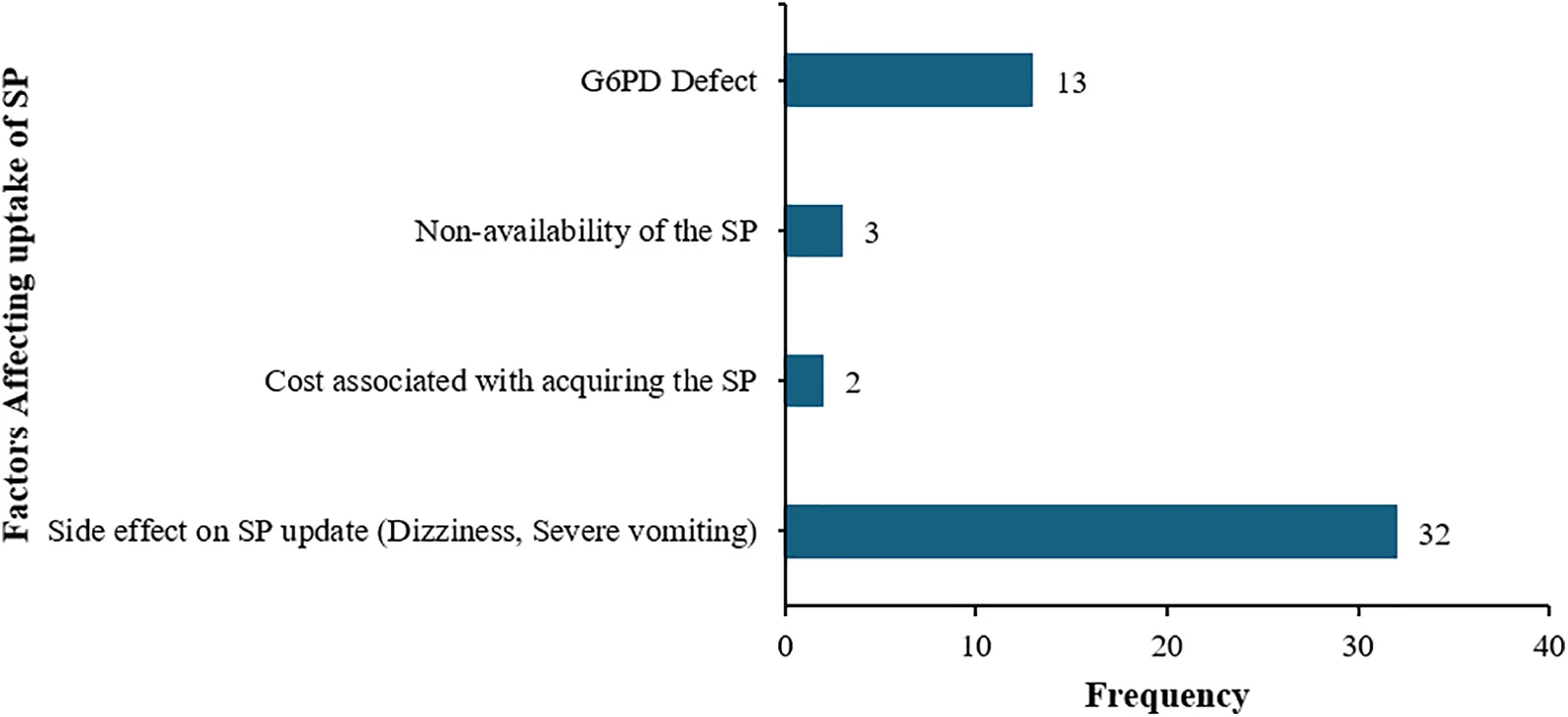
Factors influencing uptake of SP among postpartum women who did not take SP.

### Number of IPTp SP doses received during pregnancy among postpartum women

About 30.1.% (348/1155) of respondents received three doses of SP during pregnancy followed by four doses (25.3%, 292/1155). However, 7.8% (90/1155) of women received only one dose (Fig 4). Those who received at least three doses as recommended by WHO were about 69.9% (807/1155).

**Fig 4:**
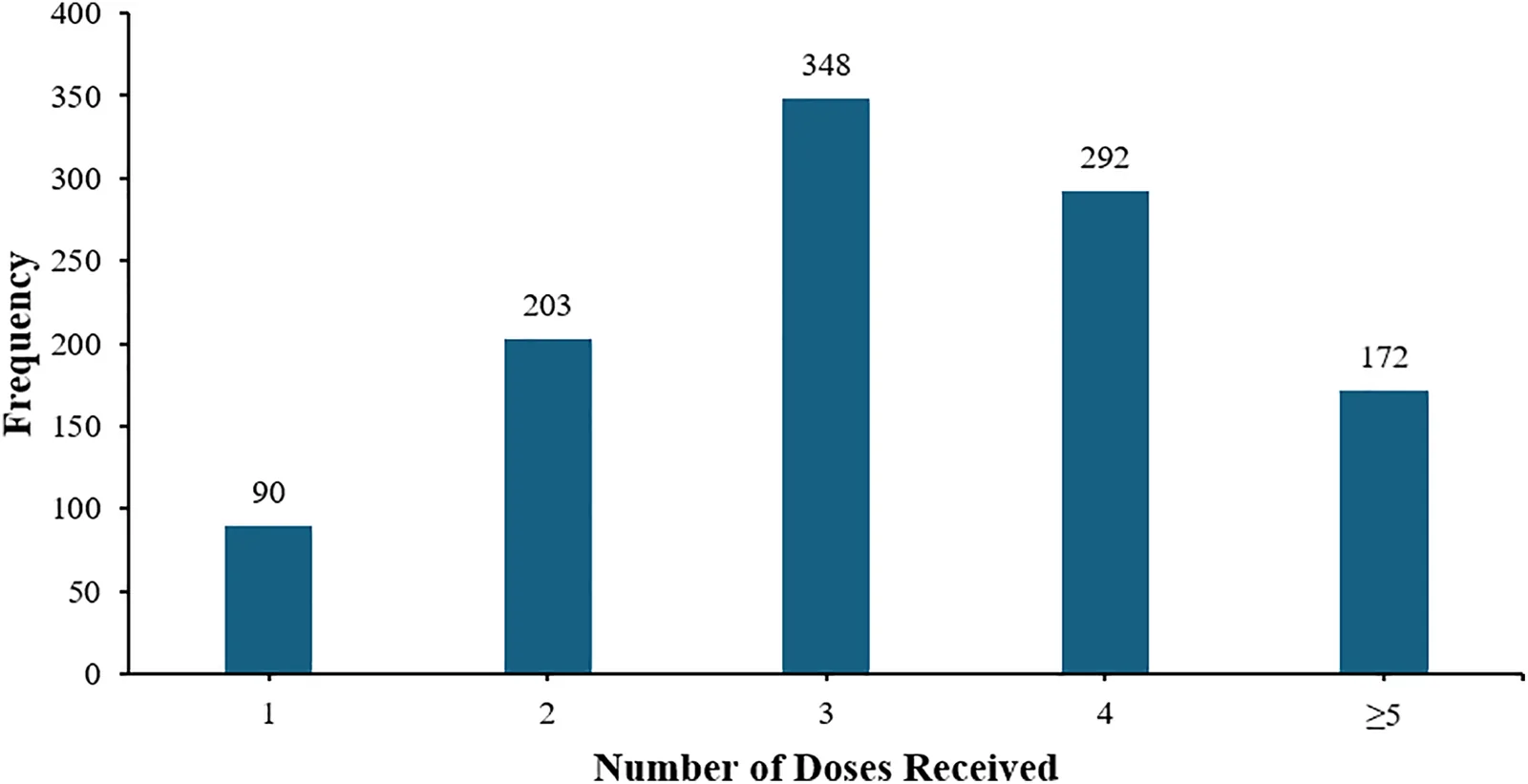
Distribution of the number of IPTp SP doses received during pregnancy among postpartum women.

### Factors independently associated with IPTp3-SP uptake

In the multivariable logistic regression analysis, several characteristics were independently associated with IPTp3-SP uptake. Women aged 10–19 years had higher odds of IPTp3-SP uptake compared with those aged 20–29 years (aOR = 2.17, 95% CI: 1.21 to 3.88; p = 0.009). Marital status was also associated with IPTp3-SP uptake. Compared with married women, single women had lower odds of uptake (aOR = 0.64, 95% CI: 0.42 to 0.97; p = 0.037), as did women who were cohabiting (aOR = 0.52, 95% CI: 0.34 to 0.79; p = 0.002). Women with a gravidity of three had higher odds of IPTp3-SP uptake compared with women with a gravidity of one (aOR = 2.67, 95% CI: 1.58 to 4.50; p < 0.001).

Health insurance status was significantly associated with uptake. Uninsured women had lower odds of IPTp3-SP uptake compared with insured women (aOR = 0.36, 95% CI: 0.20 to 0.64; p < 0.001). The number of ANC visits showed a strong association with uptake. Women who attended ANC fewer than four times had lower odds of IPTp3-SP uptake compared with those who had 10 or more visits (aOR = 0.08, 95% CI: 0.04 to 0.15; p < 0.001). No statistically significant independent associations were observed for occupation, place of residence, gestational age at booking, or other age groups, gravidity, and education (Table 3).

**Table 3:** Adjusted odds ratios for IPTp3-SP uptake from multivariable binary logistic regression (n = 1,150).

Characteristic	Adjusted OR (95% CI)	*p* value
**Age group (years)**		
10–19	2.17 (1.21–3.88)	0.009*
20–29	1.00 (Ref)	
30–39	1.09 (0.75–1.57)	0.665
≥40	1.24 (0.50–3.05)	0.646
**Educational status**		
Primary	0.35 (0.13–0.92)	0.034*
Secondary	0.51 (0.20–1.32)	0.165
Tertiary	1.00 (Ref)	
Not stated	0.35 (0.13–0.94)	0.037*
**Occupation**		
Formal employment	1.00 (Ref)	
Trading/business	0.68 (0.28–1.63)	0.388
Farming	0.46 (0.19–1.12)	0.087
Unemployed/housewife	0.46 (0.19–1.11)	0.083
Student	0.60 (0.23–1.56)	0.291
Artisan/other	0.38 (0.15–1.00)	0.051
**Marital status**		
Married	1.00 (Ref)	
Single	0.64 (0.42–0.97)	0.037*
Cohabiting	0.52 (0.34–0.79)	0.002*
Widowed	2.15 (0.20–23.09)	0.526
Divorced	0.87 (0.13–5.75)	0.881
**Place of residence**		
Urban	1.00 (Ref)	
Rural	0.97 (0.66–1.40)	0.857
**Gravidity**		
1	1.00 (Ref)	
2	1.19 (0.81–1.76)	0.379
3	2.67 (1.58–4.50)	<0.001*
4	1.83 (1.02–3.28)	0.044*
≥5	0.90 (0.51–1.59)	0.713
**Health insurance**		
Insured	1.00 (Ref)	
Not insured	0.36 (0.20–0.64)	<0.001*
**Gestational age at booking (weeks)**		
<12	1.00 (Ref)	
12–16	1.03 (0.72–1.48)	0.860
17–21	0.66 (0.40–1.10)	0.113
21–24	0.79 (0.32–1.95)	0.602
>24	0.61 (0.37–1.01)	0.054
**Number of ANC visits**		
<4	0.08 (0.04–0.15)	<0.001*
4–6	0.23 (0.13–0.43)	<0.001*
7–9	0.65 (0.34–1.25)	0.196
≥10	1.00 (Ref)	

Ref, reference category. “Not stated” under educational status is a category for missing data, not a level of education. Parity was left out of the model because of collinearity with gravidity. The model fitted significantly better than an intercept-only model (likelihood-ratio test, χ² = 279.2, df = 28, *p* < 0.001). Hosmer–Lemeshow test: χ² = 5.06, df = 8, *p* = 0.751. *p < 0.05. ANC, antenatal care; CI, confidence interval; OR, odds ratio.

### The experiences of postpartum women with IPTp-SP

#### Participants’ description.

These qualitative findings describe the experiences and perceptions of postpartum women regarding IPTp-SP in Jomoro, Prestea-Huni Valley and Ellembelle districts of the Western Region of Ghana. Twenty-eight (28) postpartum women took part in focus group discussions and in-depth interviews. Nineteen [19] participants were aged 25–39 years and nine [9] were adolescents between the ages of 17–20 years.

Four main themes emerged from the analysis: discomfort after taking SP, awareness of the benefits of SP, knowledge of the harmful effects of malaria on the unborn child, and concerns about the number and formulation of SP tablets.

### Experienced discomfort when SP was taken

Many participants reported unpleasant physical reactions after taking SP during antenatal care visits. Symptoms such as dizziness, weakness, nausea, and abdominal discomfort were commonly mentioned and sometimes created reluctance to attend clinic visits.

One participant explained, “I felt weak, dizzy and had the urge to vomit anytime I took the SP tablets during my ANC visits” (SS, 25 years). Another added, “I had continuous abdominal pains and nausea some few hours after taking the medication and this made me very uncomfortable throughout the day” (MA, 32 years).

Participants also expressed dissatisfaction with the requirement to chew the tablets during directly observed therapy. As one woman noted, “I found it very difficult chewing the 3 tablets. I think it is easy swallowing medicines, but the midwife always insisted I chew them” (AS, 37 years). In some cases, the anticipated discomfort discouraged clinic attendance. A younger participant stated, “There were times I did not want to attend ANC when my time was due to visit because of the thought of discomfort expected after taking the medicine” (DW, 19 years).

### Knowledge of the benefits of SP

Despite the discomfort reported, most women demonstrated a clear understanding of the preventive role of SP during pregnancy. Information provided during antenatal care visits was cited as the main source of this knowledge.

One respondent explained, “I was informed that the medicine served as a barrier and would protect my unborn baby from harm by the malaria parasites” (BD, 27 years). Another participant noted, “I know that the medicine protects pregnant women from malaria infection and made me healthy throughout my pregnancy” (NM, 36 years). Similarly, a participant emphasized the importance of preventing anaemia during pregnancy, stating, “I did not want to be anaemic; I learnt malaria in pregnancy can reduce the iron stores in pregnant women” (BE, 39 years).

### Awareness of the negative impact of malaria on unborn babies

Participants also demonstrated awareness of the potential adverse effects of malaria during pregnancy on the unborn child. Many linked malaria infections with complications affecting fetal growth and development.

A participant stated, “I understood that my unborn baby could be affected by the malaria parasites causing complications such as brain disorders during its development” (FC, 29 years). Another explained, “I learned that when my unborn baby is infected by the malaria parasites, it would lead to low birth weight which I did not want. I like big and strong babies” (MB, 37 years).

### Concerns about the number and formulation of SP tablets

Several participants raised concerns about the number of tablets required per dose and the repeated dosing schedule during pregnancy. Suggestions were made for modifications that could make the medication easier to take.

One adolescent participant proposed, “If possible, the 3 tablets should be put together as one tablet so that they are taken once at a go during a visit” (TA, 17 years). Another suggested a different formulation, stating, “I think it will be very helpful if the tablets are changed into capsules so that they can be swallowed instead of chewing to make it easy for pregnant women to take” (AE, 23 years).

Some participants also recommended reducing the number of doses administered during pregnancy. As one woman noted, “Can the number of doses be reduced from 5 to 3 throughout the 9 months of pregnancy?” (SQ, 42 years), while another suggested a longer interval between doses: “Authorities should consider a dose every two months than a dose each month till delivery” (CK, 38 years).

## Discussion

This study examined the uptake of IPTp3-SP among postpartum women in three rural districts in the Western Region of Ghana and explored the contextual factors influencing utilization through women’s lived experiences.

The study found that 96% of postpartum women received at least one dose of IPTp-SP during pregnancy, suggesting generally high coverage and acceptance of the intervention within the study districts. This finding is comparable to the 98.5% coverage reported by Ibrahim et al. (2017) in the Sunyani Municipality of Ghana [15]. The quantitative findings further showed that most women attended four or more ANC visits, which likely contributed to the high uptake observed. However, qualitative findings revealed that high uptake did not necessarily translate into positive experiences with SP administration. Many women described adverse reactions such as dizziness, nausea, abdominal discomfort, weakness and vomiting after taking SP. These experiences help explain why a minority of women failed to initiate or complete IPTp-SP despite attending ANC services. Similar findings were reported by Dun-Dery et al. (2021), who observed that unpleasant side effects and previous negative experiences reduced willingness to continue SP use during pregnancy [17].

This therefore suggests that while programmatic access to IPTp-SP may be high, experiential barriers continue to influence adherence behaviours. Some participants reported intentionally delaying or missing ANC visits due to fear of discomfort after SP administration. This finding is important because it demonstrates that utilization cannot be understood solely from coverage indicators without considering women’s perceptions and experiences during service delivery.

The study also identified age, educational status, marital status, gravidity, health insurance status and number of ANC visits as independent predictors of IPTp3-SP uptake. These findings indicate that IPTp3-SP utilization in this population was influenced by both individual characteristics and contact with maternal health services.

The number of ANC visits showed the strongest and most consistent association with IPTp3-SP uptake. This finding is consistent with several Ghanaian studies. Owusu Boateng and Anto reported that women who attended at least four ANC visits were more likely to receive three or more doses of SP, while Addai-Mensah et al found a strong association between regular ANC attendance and completion of the recommended IPTp-SP doses [21,22]. Similarly, the Kintampo study found that attending ANC at least five times was independently associated with receiving three or more doses (OR = 2.83, 95% CI: 2.64 to 3.03) [23]. The qualitative findings provide additional context for this association. Participants described ANC visits as important opportunities to receive SP and obtain information from midwives about its benefits. Some women, however, reported delaying or missing ANC because of anticipated discomfort after taking SP. This suggests that frequent ANC attendance increases opportunities for supervised SP administration and repeated counselling, whereas missed visits can interrupt the sequence of doses. Similar findings have been reported in Ghana, where the number of maternal ANC contacts was associated with IPTp-SP uptake and late initiation of ANC was identified as a barrier. The Ghanaian ethnographic study by Dun Dery et al. found that regular ANC attendance, provider practices and women's trust in health workers influenced continued IPTp-SP use [17].

Health insurance status was also independently associated with IPTp3-SP uptake. Uninsured women had lower odds of receiving IPTp3-SP than insured women. This finding is consistent with Ghanaian evidence showing that insurance status is associated with maternal service utilization and IPTp3-SP uptake. In a study of women with obstetric referrals, health insurance status, ANC attendance, marital status and facility type significantly influenced the number of IPTp doses received [24]. Similarly, a study conducted in predominantly rural areas of Ghana found that possession of health insurance was positively associated with attending ANC at least four times, particularly among women with lower socioeconomic status [25]. Although IPTp-SP itself is delivered through ANC services, insurance may therefore act as a marker of broader engagement with maternal health services and ability to maintain regular contact with care.

Age was independently associated with IPTp3-SP uptake. Adolescents aged 10–19 years had higher odds of IPTp3-SP uptake than women aged 20–29 years (aOR = 2.17, 95% CI: 1.21 to 3.88). This finding differs from the Kintampo study, in which women aged 20 years or older were more likely to receive three or more doses after adjustment [23]. The difference may reflect variations in study population, setting, sample composition and the definition of optimal IPTp uptake. The qualitative findings indicate that adolescent participants were aware of the potential benefits of SP and described direct provider supervision during ANC. However, the relatively small number of adolescents in the qualitative component means that this explanation should be interpreted cautiously.

Educational status was also independently associated with IPTp3-SP uptake. Women with primary education had lower odds of uptake than women with tertiary education. This finding agrees with Ghanaian studies showing that educational attainment is associated with IPTp3-SP uptake. Addai-Mensah et al. found higher odds of completing the recommended IPTp-SP doses among women with tertiary education, while the Kintampo analysis also identified education as an independent predictor of receiving three or more doses [22,23]. The qualitative findings suggest that provider counselling may help reduce some differences in knowledge because participants across educational groups reported receiving information from midwives. Nevertheless, the lower uptake among women with primary education suggests that repeated and understandable counselling may remain important.

Marital status was independently associated with IPTp3-SP uptake. Compared with married women, single women had lower odds of uptake, as did women who were cohabiting. This finding is consistent with the Kintampo study, where married women were more likely to receive three or more doses after adjustment [23]. Similarly, a study in the Gushegu District found single status to be associated with inadequate IPTp uptake [26]. One possible explanation is that marital status may reflect differences in social support, household resources and support for continued ANC attendance. Evidence from rural Ghana also shows that single and cohabiting women were less likely to achieve at least four ANC visits [25]. However, these mechanisms were not directly examined in the present study and should therefore be regarded as possible explanations rather than causal conclusions.

Gravidity was another independent predictor of IPTp3-SP uptake. Women with three pregnancies had higher odds of uptake than primigravidae, while women with four pregnancies also had higher odds. This finding is broadly consistent with evidence that pregnancy experience can influence IPTp utilization, although the direction and strength of the association vary between studies. For example, the Kumasi study found lower odds of completing the recommended IPTp doses among women with more than four children, while the Gushegu study identified reproductive history as a factor associated with IPTp uptake [22,26]. Differences may relate to the way gravidity, parity and number of living children were categorized across studies. Women with previous pregnancies may also have greater familiarity with ANC procedures and SP administration, although this explanation was not directly tested in the present study.

The study found no independent association between IPTp3-SP uptake and occupation, place of residence or gestational age at booking. The absence of an association with residence differs from the Kintampo study, where place of residence was independently associated with uptake [23]. This difference may partly reflect the predominantly rural setting of the present study, where women may have had more comparable access to ANC services. Similarly, the absence of an association with gestational age at booking differs from studies in which timing of the first ANC contact was associated with IPTp uptake [17]. The difference may reflect variation in ANC attendance patterns and service delivery across study settings.

Despite the high uptake, the qualitative findings identified important experiential barriers to continued use of SP. Participants reported dizziness, nausea, weakness, abdominal discomfort and vomiting after taking SP. Some also disliked chewing the three tablets during directly observed therapy. These experiences may help explain why high coverage did not translate into universal completion of IPTp-SP. Previous qualitative research in Ghana has similarly documented concerns about SP administration and adverse experiences. In Northern Ghana, participants reported discomfort associated with SP, including vomiting, while a more recent qualitative study in southern Ghana found that women commonly experienced nausea and dizziness but generally continued SP because they perceived benefits for themselves and their unborn babies [12,27].

Although general awareness of malaria transmission and prevention was high in the quantitative analysis, interviews revealed important gaps in detailed understanding of IPTp-SP. Many participants knew malaria could harm pregnancy and unborn babies, yet several could not identify SP by name or clearly explain its preventive role. Some women reported taking the medication primarily because it was administered during ANC visits rather than through informed understanding of its purpose. Similar observations have been reported by Nwaefuna et al. (2023) and Aberese-Ako et al. (2026), who found that awareness of malaria in pregnancy did not always correspond with adequate knowledge of IPTp-SP [11,12].

The findings from this study therefore highlights an important distinction between compliance and informed adherence. While women generally complied with SP administration, the qualitative findings suggest that adherence was often dependent on provider direction and continued supervision. This may present challenges for long-term sustainability if provider communication is inadequate or if women experience adverse reactions without proper counselling and reassurance.

The study additionally identified concerns regarding the formulation and administration of SP. Some participants disliked chewing the tablets during directly observed therapy and preferred swallowing or alternative formulations such as capsules. Others expressed concern about the number of tablets and repeated monthly dosing schedule. Similar concerns regarding bitterness, side effects, and tablet burden have been documented in studies from Uganda and other malaria endemic settings [28]. These findings emphasize the need for more patient-centered approaches within ANC services, including improved counselling on expected side effects, supportive communication during directly observed therapy, and reassurance regarding the benefits of IPTp-SP.

Addressing these experiential and behavioural factors alongside routine service delivery may further strengthen IPTp-SP utilization and improve maternal and neonatal health outcomes in malaria endemic settings.

### Limitations and strengths of the study

This study has several limitations. First, the cross-sectional design limits the ability to establish temporal relationships between the identified factors and IPTp-SP uptake; therefore, the observed associations should not be interpreted as causal relationships. Second, IPTp-SP doses were self-reported and relied partly on participants’ recollection of their ANC experiences, introducing the possibility of recall bias and misclassification of IPTp-SP uptake. Self-reported information may also have been influenced by social desirability bias, particularly where participants perceived that reporting adherence to recommended IPTp-SP doses was expected. This was however cross-checked from ANC records at their respective ANC clinics as much as possible.

Third, participants were recruited from selected health facilities, which may have introduced selection bias and may have excluded women who did not attend ANC or who sought care outside the participating facilities. Consequently, the findings may not be fully generalisable to all pregnant or postpartum women in Ghana, particularly those in urban settings or regions with different health-service and sociodemographic characteristics. The study was conducted in rural communities in the Western Region, further limiting the generalisability of the findings beyond rural Western Ghana.

Although place of residence was captured, the study did not specifically assess the burden and cost of travelling to ANC facilities, including distance, transportation availability, travel time, and transport expenses. These factors may influence the ability of women in rural communities to attend ANC regularly and, consequently, their opportunities to receive IPTp-SP. Future studies should incorporate these access-related factors and, where possible, corroborate self-reported IPTp-SP uptake with ANC records or other routine documentation.Nonetheless, the study’s strength lies in its integration of both quantitative and experiential perspectives, providing a comprehensive understanding of IPTp-SP uptake dynamics in a real-world rural context.

## Conclusion

This study demonstrates that optimal uptake of IPTp-SP among postpartum women in rural districts of the Western Region of Ghana is high, about seven in ten women took three or more doses of SP during pregnancy. Uptake was largely independent of socio-demographic and obstetric characteristics, particularly age, education, marital status, gravidity, health insurance status, and number of ANC visits. However, Women with fewer ANC visits had lower odds of receiving three or more doses. Despite high coverage, qualitative findings revealed important experiential barriers, including perceived side effects, challenges with drug administration, and limited understanding of IPTp-SP may affect adherence and completion of recommended doses. Strengthening ANC attendance and counselling on the benefits, administration, and expected side effects of IPTp-SP may help address these barriers and support sustained uptake.

### Recommendations

At the national level, programme managers should prioritize strategies that promote early initiation and consistent attendance of antenatal care (ANC), given its strong association with IPTp-SP uptake. At the sub-national level, Regional and District Health Directorates should intensify community-based health education and outreach to improve ANC retention and address gaps in service utilization. Additionally, health facility managers and frontline ANC providers should enhance patient-centered counselling by clearly communicating the benefits of IPTp-SP, addressing concerns about side effects, and improving provider–client interactions to foster trust and adherence. These coordinated efforts will be critical to optimizing IPTp-SP uptake and improving maternal and neonatal health outcomes in Ghana and other malaria-endemic settings.

## Abbreviations

ANC: Antenatal care
CWC: Child welfare clinic
DOT: Directly observed therapy
GHS: Ghana Health Service
IPTp: Intermittent preventive treatment in pregnancy
IPTp-SP: Intermittent preventive treatment in pregnancy with sulfadoxine–pyrimethamine
MIP: Malaria in pregnancy
NMEP: National Malaria Elimination Programme
ODK: Open Data Kit
SP: Sulfadoxine–pyrimethamine
SSA: Sub-Saharan Africa
WHO: World Health Organization
